# Supplementary material for: European-wide forest monitoring substantiate the neccessity for a joint conservation strategy to rescue European ash species (Fraxinus spp.)
Source: Sci Rep. 2022 Mar 19;12:4764. doi: 10.1038/s41598-022-08825-6 (PMC8934346; doi:10.1038/s41598-022-08825-6)
Supplement: Supplementary file 1 — Supplementary Information. [file 41598_2022_8825_MOESM1_ESM.pdf]

Supplementary Information S1

Tests for zero slope for Cox-proportional hazard models

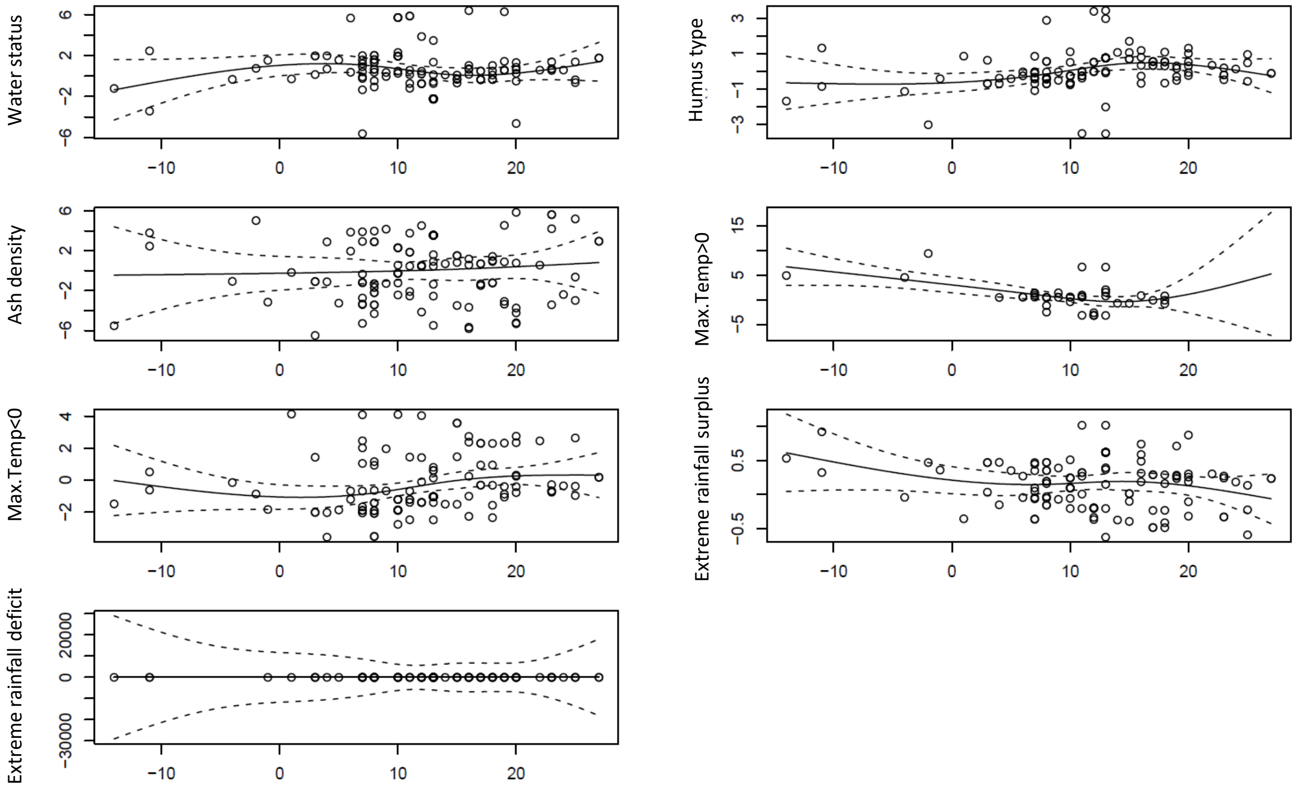

Estimated cumulative regression functions with 95% pointwise confidence intervals based on Aalen’s additive model

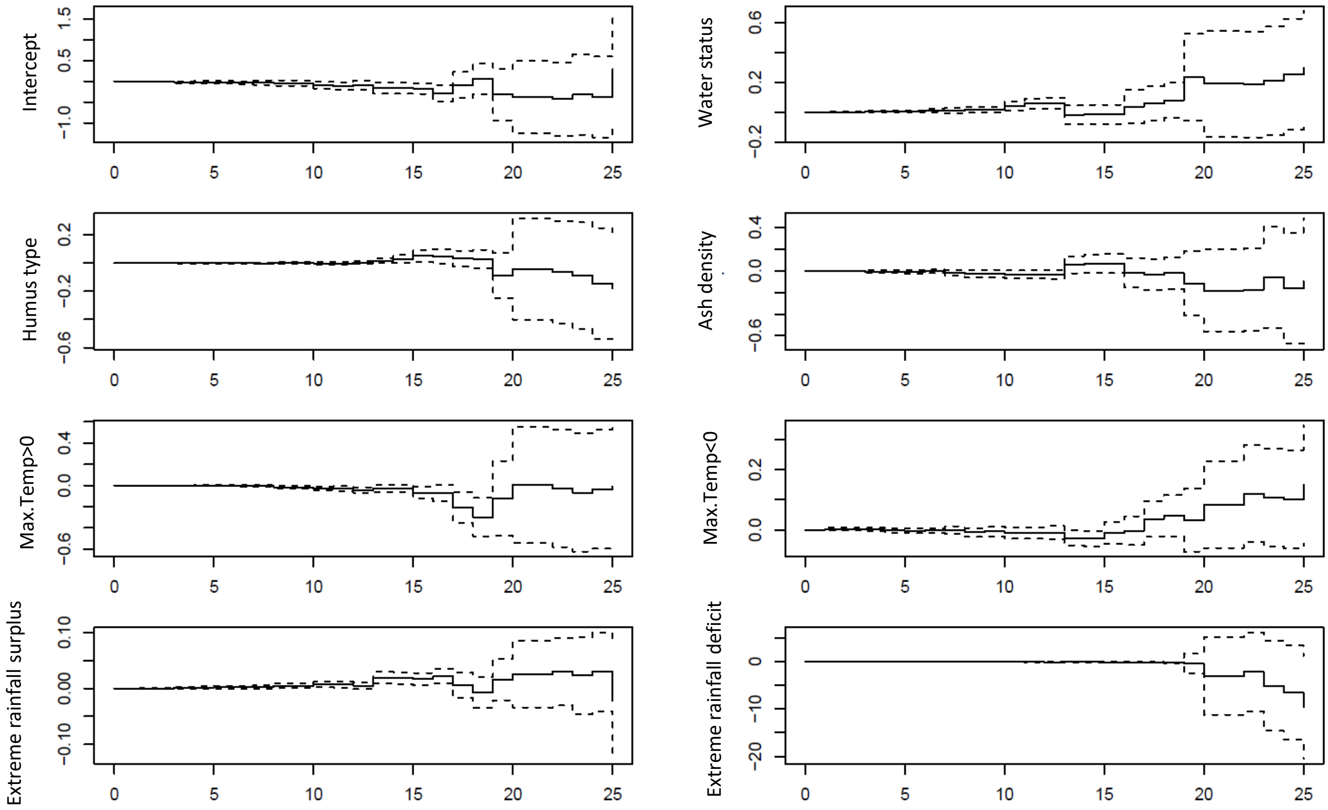

Supplementary Information S2

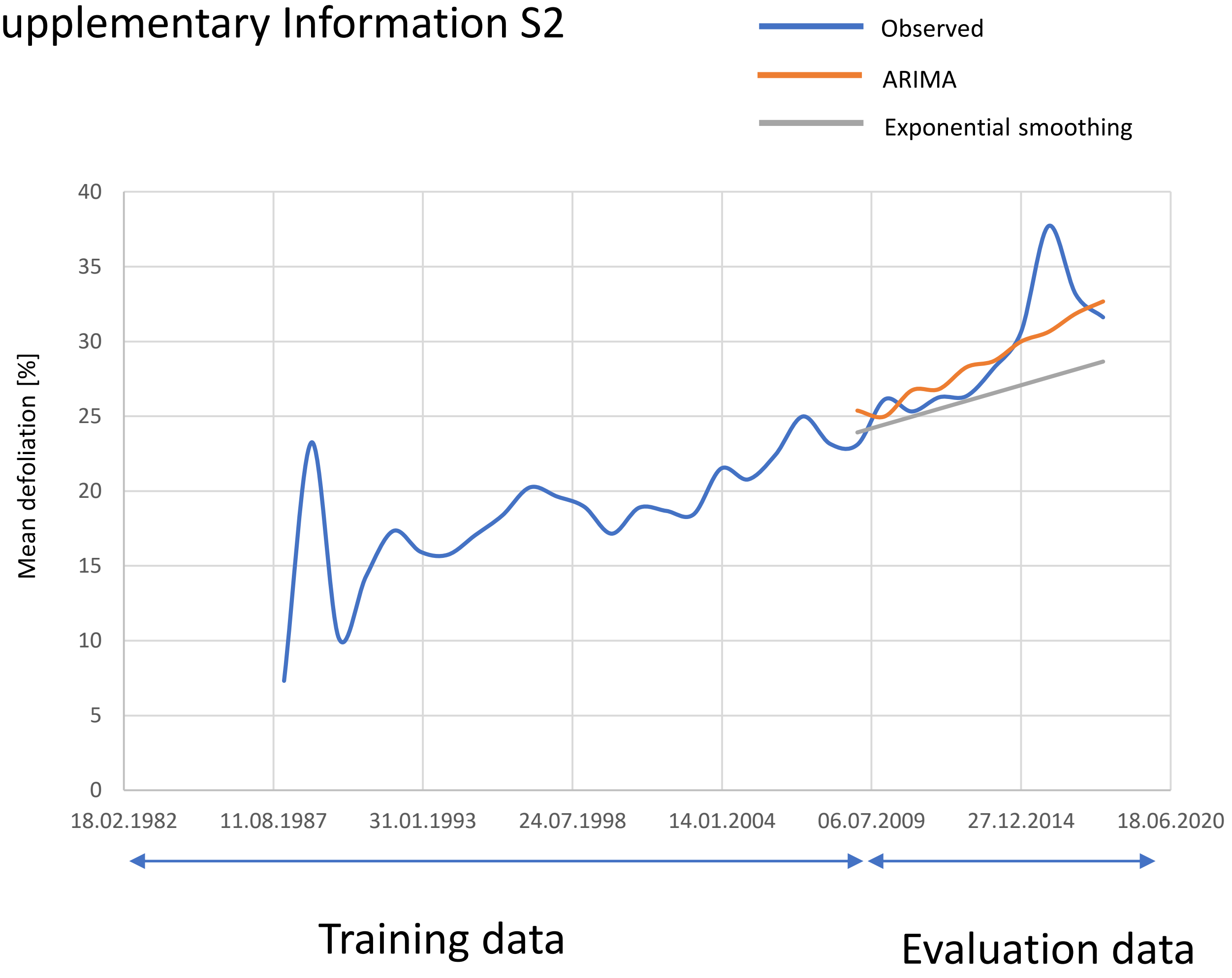

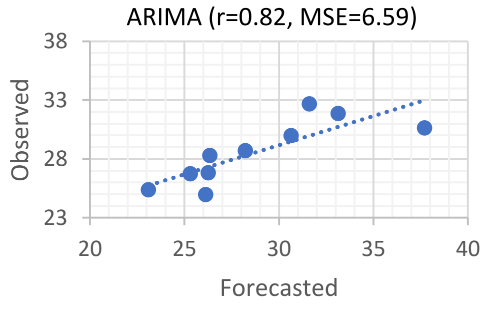

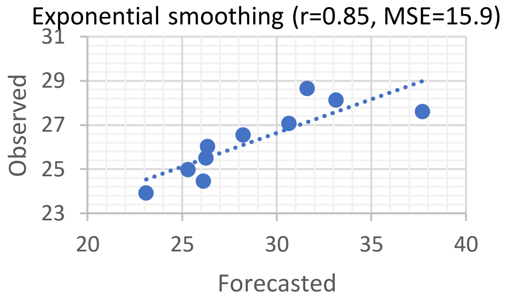

Supplementary Information S3

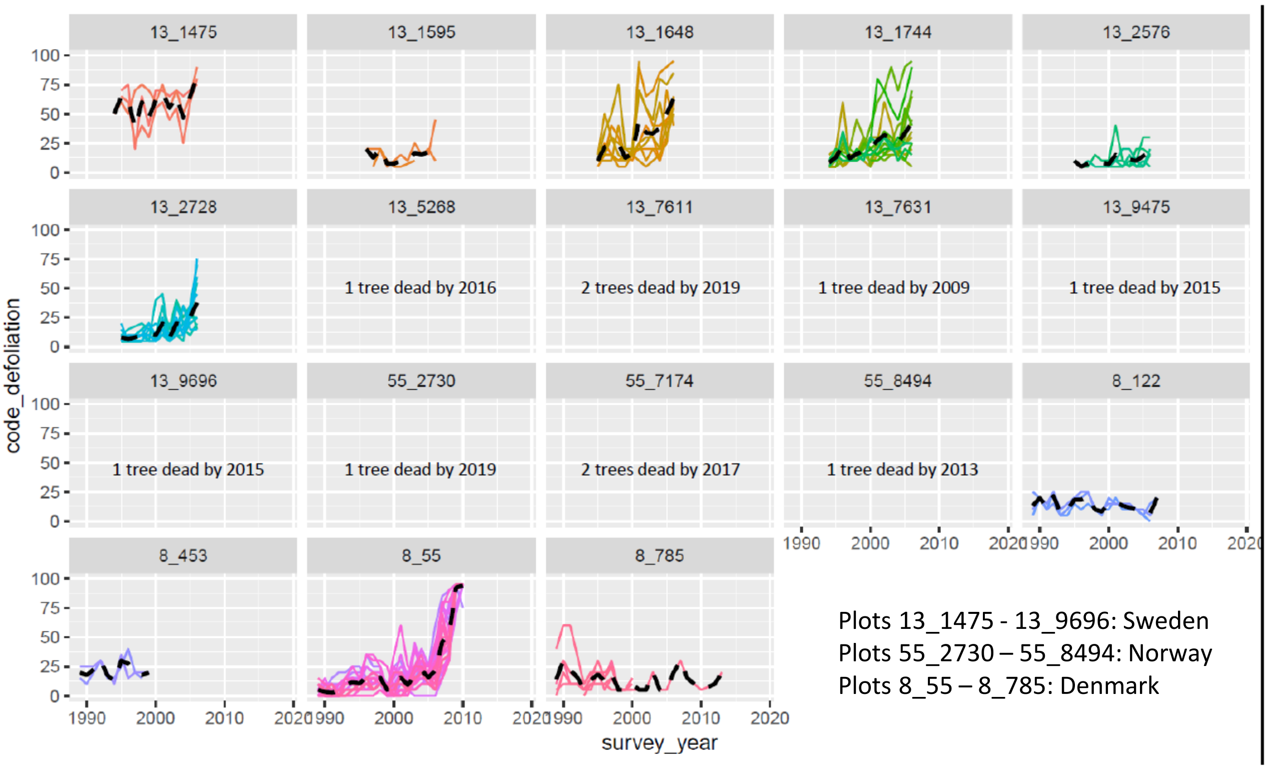

Plots 13_1475 - 13_9696: Sweden
Plots 55_2730 – 55_8494: Norway
Plots 8_55 – 8_785: Denmark
